# 2491: Parental concerns about child participation in s reflect a need to move beyond traditional notions of trust and race

**DOI:** 10.1017/cts.2017.139

**Published:** 2018-05-10

**Authors:** Jennifer Erves, Tilicia Mayo-Gamble, Consuelo Hopkins Wilkins

**Affiliations:** Vanderbilt University, Nashville, TN, USA

## Abstract

OBJECTIVES/SPECIFIC AIMS: The objective of this study was to identify factors influencing parental willingness of adolescent participation in clinical trials. METHODS/STUDY POPULATION: We applied community engaged research principles to conduct a theory-based, cross-sectional study of parental willingness. Parents (N=307) were given a survey from November 2014 to April 2015. Factors influencing parental willingness were identified using binary logistic regression. SPSS version 22.0 was used to perform analyses, and p<0.05 was considered statistically significant. RESULTS/ANTICIPATED RESULTS: The most impactful factor on willingness was Advantages of Adolescent Clinical Research (p = .001), followed by Disadvantages of Clinical Research (p = .006), Knowledge of Adolescent Clinical Trials (p = 0.029), and Perceived Health Status of Adolescent (p = .036). In further exploring the influence of Perceived Advantages and Perceived Disadvantages, “My child will do something to help others.” (p = .026) and “My child is too young to participate in a clinical trial.” was the only significant Perceived Disadvantage (p = .001) were significantly associated with parental willingness. DISCUSSION/SIGNIFICANCE OF IMPACT: Improving parental knowledge and understanding of adolescent clinical trials, the advantages and disadvantages of adolescent participation, and the health status requirements for child participation are important factors to address when influencing parental willingness to allow adolescents to participate in clinical trials. Recruitment strategies that incorporate this information could improve future adolescent participation in clinical trials, ultimately promoting adolescent health and disease prevention.

